# Molecular Biomarkers of Adult Human and Dog Stress during Canine-Assisted Interventions: A Systematic Scoping Review

**DOI:** 10.3390/ani12050651

**Published:** 2022-03-04

**Authors:** Jaci Gandenberger, Erin Flynn, Em Moratto, Ashley Wendt, Kevin N. Morris

**Affiliations:** Institute for Human-Animal Connection, Denver, CO 80210, USA; jaci.gandenberger@du.edu (J.G.); erin.fleming@du.edu (E.F.); em.moratto@du.edu (E.M.); ashley.wendt@du.edu (A.W.)

**Keywords:** human–canine interactions, animal-assisted interventions, stress biomarkers, molecular biomarkers

## Abstract

**Simple Summary:**

Interactions between people and dogs may lower participants’ stress levels. However, this is a fairly new area of research and there has not been a review of what we know across studies. We examined the existing research and found that human–dog interactions consistently improve some indications of human stress levels and don’t seem to negatively affect dogs. However, we need to do more research to gain a better understanding of the impacts on people and dogs with a wider lens that looks at more markers of stress.

**Abstract:**

Positive relationships, including those between humans and other animals, particularly dogs, may be a way to reduce stress in humans. However, research into this area is relatively new, and a comprehensive review of the impacts of these interactions on humans and dogs has not been conducted. A scoping review of the scientific literature was conducted to explore what is known about the impacts of canine-assisted interventions on molecular biomarkers (e.g., cortisol and oxytocin) and associated measures (e.g., heart rate and blood pressure) of human and canine stress. As reported across 27 identified studies, canine-assisted interventions have consistently been demonstrated to elicit positive changes in human stress markers, and typically do not cause negative impacts on the studied canine stress markers. However, results were inconsistent across measures of stress. For example, in humans, it was common for a study to show improvements to cortisol levels but no change to self-reported stress, or vice versa. Many of the reviewed studies also had significant methodological issues, such as not aligning the timing of sample collections to when the analyzed stress biomarkers could be expected to peak. More rigorous research should be conducted on the impacts of canine-assisted interventions on a wider range of stress biomarkers.

## 1. Introduction

In 2020, the American Psychological Association warned that the rise in stress levels across the United States pointed to a “mental health crisis that could yield serious health and social consequences for years to come” [1]. Many disorders, including hypertension, diabetes, asthma, and arthritis, originate from or can be aggravated by acute or chronic stress [2,3]. Stress affects many physiological functions, including the immune, nervous, and endocrine systems [4]. Chronic stress can also lead to physiological maladaptations that have been linked to long-term adverse health effects, including depression, anxiety, allergies, cancer, and post-traumatic stress disorder (PTSD) [5,6].

One strategy to reduce stress is through positive relationships, including with a companion animal, such as a dog. For example, Morales-Jinez et al. (2018) compared two groups of older adults who lived with and without canine companions and found reduced levels of cortisol and total cholesterol, biomarkers associated with stress, in participants who owned dogs [7]. These differences are likely due in part to lifestyle differences: dog owners have been shown to exercise more often and sleep better [8]. However, the presence of a dog also has immediate impacts on stress responses. For example, Krause-Parello and Gulick (2015) found that children who were accompanied by a therapy dog while participating in forensic interviews exhibited lower stress levels, as assessed by heart rate (HR) [9]. Other research has found that interactions with a dog may be more successful than interactions with a close friend at moderating people’s stress levels [10,11,12]. These findings have encouraged the development of animal-assisted interventions (AAIs), sometimes referred to as animal-assisted activities (AAAs) or animal-assisted therapies (AATs), to enhance the physical, cognitive, behavioral, and/or social–emotional functioning of participants through interactions with a non-human animal [13].

How these AAIs impact the stress levels of humans and dogs involved is currently unclear and understudied [14,15,16]. Preliminary research indicates that both acute and chronic stress levels may be synchronized in dogs and their owners [17,18,19]. Some studies have found that dogs have higher levels of cortisol following AAI sessions, while other research found decreased or unchanged cortisol levels [20,21,22,23,24]. However, even those studies that showed elevated cortisol in dogs after AAIs could not discern if the dogs’ arousal was due to positive excitement or negative stress, because cortisol alone cannot differentiate between distress and eustress [14,21,23,25]. Researchers have cautioned that, due the complexity of stress-response processes, no single metric has been found to be sufficient to understand the presence or extent of stress in humans or other animals [26,27,28].

To date, no systematic review has been conducted on the available evidence regarding AAIs’ impacts on humans and dogs as assessed by a broad array of stress biomarkers. The purpose of this systematic scoping review is to determine what is known about the use of molecular biomarkers (e.g., cortisol and oxytocin) and related physiological (e.g., blood pressure (BP) and HR) and subjective (self-report) measures to study adult human and canine stress in the context of AAIs. By examining the biological pathways and other indicators of stress that are impacted by human–animal interactions during AAIs, researchers can develop a deeper understanding of the mechanisms that underlie the outcomes of these interactions, develop tools to measure their impacts more directly and accurately, and identify the most effective approaches to utilize human–animal interactions to support human and canine well-being.

## 2. Materials and Methods

This systematic scoping review assessed research that used molecular biomarkers to measure human and canine stress during AAIs. Systematic scoping reviews aim to evaluate the literature in terms of the volume, nature, and characteristics of the primary research in a selected area of interest and can be especially useful when the topic has not yet been extensively reviewed, as in this case [29]. A rigorous systematic scoping review of quantitative and mixed-methods research was conducted in accordance with the Preferred Reporting Items for Systematic Reviews and Meta-Analyses extension for scoping reviews (PRISMA-ScR) [30]. No ethical approval was required for this review.

The research question was developed through discussion among team members engaged in human–animal interaction research and with expertise in molecular biology. The overarching question was asked: “what is known about the use of molecular biomarkers and associated physiological and subjective measures of stress responses to study adult human and canine stress, both acute and chronic, in the context of human–canine interactions?” To add specificity to this inquiry, three sub-questions were used:

1. Which molecular biomarkers have been used to measure the effects of AAIs on human and/or dog stress?
2. What stress-related outcomes have been found for humans and canines?
3. In studies that measure molecular biomarkers of stress, do any also include physiological measures or subjective or behavioral assessments of the same or related outcomes? If so, what measures were used and what outcomes were found?

### 2.1. Identifying Relevant Studies

A three-step search strategy was used to conduct the scoping review. First, an initial limited search of two online databases relevant to the topic was completed, during which newly discovered search terms or databases were added to the protocol. Second, a search was completed that used all identified keywords across all included databases. Lastly, reference lists of the sources selected for inclusion were searched until saturation was reached. The following electronic databases were searched: Web of Science social science and science citation indices, PubMed, PsycInfo, Agricola, Biological Abstracts, Google Scholar, Academic Search Complete, Human–Animal Bond Research Institute (HABRI) Central, ERIC, Sociological Abstracts, and Opengrey.org. In addition, conference abstracts published between 2015 and 2020 were reviewed from conference proceedings of the International Association of Human–Animal Interaction Organizations, International Society for Anthrozoology, American Psychological Association, and the Society for Social Work and Research. 

Content experts on the research team worked in consultation with a social-sciences research librarian to develop a search strategy using concepts and keywords to describe human–canine interaction, stress, and biomarkers. One example of the exact search string was (“human–canine” OR “human–dog” OR “canine-assisted” OR “dog-assisted” OR dog OR canine) AND (interaction* OR intervention* OR therap* OR relationship* OR connection* OR treatment*) AND (stress* OR anxiety OR “panic disorder*” OR “adjustment disorder*” OR trauma*) AND (biomarker* OR “biological marker*” OR cortisol OR oxytocin OR protein OR molecular). The complete search strategy with versions of this search string modified for different databases is available upon request.

Eligibility criteria dictated that research articles included AAIs as a component of the intervention or experimental condition; that stress in people and/or canines was measured by using molecular biomarkers; that the study focused on the impact of an AAI on stress or a type of human anxiety disorder included in the DSM-V, with the exception of reactive attachment disorder and disinhibited social engagement disorder; and/or the impact of interactions on stress in dogs was assessed. No date restrictions were used to allow for the most comprehensive capture of previous methods used. Studies may have involved adult participants of any race, gender identity, socioeconomic status, sexual orientation, ethnicity, ability level, religion, immigration status, or nationality; and dogs of any age, sex, or breed. Studies with only participants who were children or older adults (i.e., over 65) were excluded due to limitations in the resources available to conduct this review. For inclusion, studies had to have been published in English, Spanish, French, German, or Hawaiian and be either primary research, conference abstract, dissertation, or thesis.

Studies were excluded that focused only on the human participants’ subjective experience of stress (i.e., self-report of emotions or cognition), did not include a specific stress event or stress-related diagnosis, measured only physiological outcomes but not molecular biomarkers, or took place in veterinarian settings and measured only dogs’ molecular and physiological responses to non-human factors in the environment. Due to the limited resources available to conduct this search, only the first 100 returned sources in the target languages, as sorted by relevance, were screened for each database. Similarly, books, unpublished research, research reports, and government reports were not included, nor were key journals or recommendations from outside professional networks. Search results were documented in Zotero (Corporation for Digital Scholarship, Inc., Vienna, VA, USA) and duplicates were removed.

### 2.2. Study Selection

Screening was conducted in two stages. First, 25 returned sources were randomly selected and screened by the full research team, using the eligibility criteria. The team met to discuss discrepancies and, when needed for screening accuracy, made modifications to the eligibility criteria. Once the reviewer agreement surpassed 75%, the team divided and screened the remaining articles. At least one researcher who was fluent in the article’s language screened the articles not available in English. Articles were first screened according to the title and abstract to remove articles that were ineligible. The full text of the article was reviewed when information in the title and abstract was insufficient to determine if the article met inclusion criteria for the present study. Articles for which it remained unclear whether they met inclusion criteria were discussed by the full team. For excluded sources, reasons for exclusion were tracked in a spreadsheet (available upon request).

### 2.3. Data Extraction and Synthesis

A study-specific data charting form was created to capture data of interest across 5 categories: general study design (i.e., type of design, human and dog demographics, and experimental condition), intervention description (i.e., name, frequency, structure, and length), outcome measures (i.e., type of molecular biomarker(s) used for humans and dogs, type of physiological response(s) measured for humans and dogs, timing of samples, and subjective and behavioral measures of stress), key outcomes, and study limitations. Once studies were selected, two reviewers pilot-tested the data charting form for the same three articles and compared their entries for accuracy. The form was revised where needed to ensure that all targeted data were captured according to the review questions. Reviewers then extracted and analyzed data for the remaining articles to generate frequency counts of key variables. A narrative synthesis was conducted to investigate the similarities and differences between findings of different studies and explore patterns in the data according to Cochrane guidelines for narrative synthesis [31].

## 3. Results

### 3.1. Overview of Search Results

**Figure 1:**
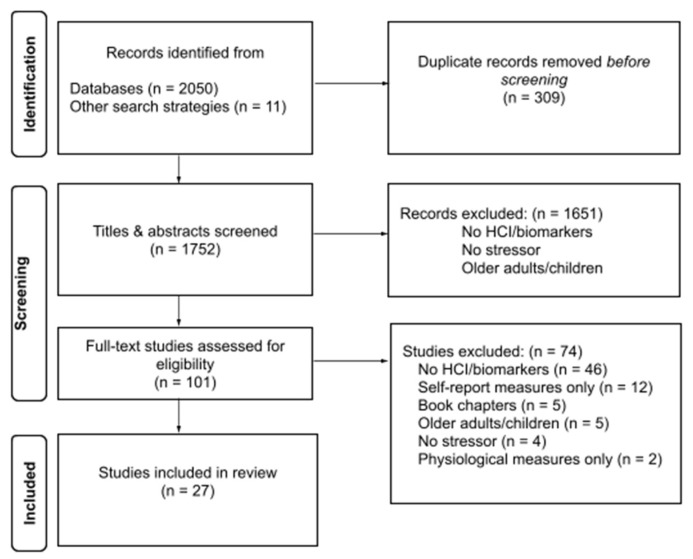
PRISMA flow diagram showing reference screening strategy over time and common reasons for exclusion.

A total of 2050 references were screened for eligibility. Source selection included articles in English, German, French, and Spanish. No Hawaiian-language records were returned during the search. In total, 1654 English, 196 French, 172 German, and 28 Spanish were initially identified. After the removal of duplicates, titles and abstracts were screened for 1752 remaining records. Of these, 1651 records were excluded. Next, full texts of 101 articles (100 English and 1 Spanish) were reviewed, and the Spanish and 73 English articles were excluded. Twenty-seven English articles were eligible for inclusion (see Figure 1). The included articles each had data extracted regarding their study population, design, and findings (see Table 1).

### 3.2. Study Characteristics and Key Results: Humans

Eighteen of the 27 studies examined molecular biomarkers in the context of human participants’ responses to AAIs [26,32,33,35,37,38,40,43,44,45,46,47,48,49,50,51,52,55]. However, none studied the impacts of AAIs within general adult populations that had a relatively even balance of men and women and members of various racial and ethnic groups. Most of the studies focused on specific populations or circumstances, such as hospitalized patients [37,38,45,46,52], parents of autistic children [40], or veterans diagnosed with PTSD [55]. In the studies that reported relevant demographic data, White participants and women were both overrepresented. No studies reported having transgender or non-binary participants. 

Each of the 18 studies mentioned above found that AAIs had a significant positive impact on at least one measure of stress. However, results often varied in the same study across assessment methods. For example, cortisol was often studied alongside HR and/or BP [12,32,35,37,45,48,49,52,54], but changes between those physiological measures and cortisol were often not correlated. Of the nine studies that measured salivary cortisol in tandem with HR, five found substantially different outcomes between the two measures (e.g., AAI groups showing lower cortisol but similar HR compared to baseline or control, or vice versa) [12,32,35,37,45]. BP and salivary cortisol were measured together in five studies [32,45,48,49,52], and only three of those studies found similar results across both measures [48,49,52]. The studies measuring self-reported stress alongside molecular biomarkers [12,22,26,32,33,35,37,38,40,43,44,45,46,47,48,49,52,55] also had mixed findings: half found different outcomes across the two measures [12,32,33,35,37,45,48,49,52], while four saw improvements in both self-report and molecular biomarkers of stress [38,43,44,55]. 

The timing of sample collection appeared to have an important impact on results. Significant changes to salivary cortisol were only found in one [44] of the six studies [35,37,39,44,52,54] in which samples were only taken less than 30 min post-intervention. The only exception featured the longest intervention, lasting 70 min, which may be why it still found a significant cortisol impact. Ten studies sampled salivary cortisol from humans at least 30 min post-AAI [12,22,32,43,45,47,48,49,50,53], of which all but two [48,49] found that the presence of a dog resulted in significantly lower cortisol levels. 

Researchers reportedly found collecting and measuring molecular biomarker samples challenging for several reasons. Two studies attributed their lack of statistically significant cortisol results to their failure to collect cortisol 45 min post-intervention, to accommodate for cortisol lag time and optimal time of cortisol collection [35,37]. Most others did not report when they took samples or the effects that time of day and the associated peaks and drops of biomarkers may have had on their results. Only three studies [12,45,49] mentioned sampling time of day as a factor in their study design, even though cortisol, sAA, and IgA are all known to fluctuate throughout the day [56,57,58]. Similarly, few studies controlled for factors known to impact cortisol levels, such as exercising beforehand and eating or drinking before sampling, which may have impacted their results [59].

### 3.3. Study Characteristics and Key Results: Dogs

Nine of the 27 studies examined stress-related molecular biomarkers in dogs as a response to AAIs [26,34,36,39,41,42,43,53,54]. All but one used certified therapy, facility, or service dogs, while the remaining study focused on dogs residing in an animal shelter [47]. Most of the studies used a variety of dog breeds or did not provide breed information. Only eight studies stated whether the dogs had been spayed or neutered [22,35,36,39,41,42,51,54], and five of those had a mix of dogs that were and were not sterilized [22,39,41,42,54]. 

Of the nine studies that examined canine stress, only one found statistically significant increases to participating dogs’ stress levels as a result of participating in an AAI [39]. Only three studies looked at both molecular biomarkers and physiological measures of canine stress [36,39,54], making it difficult to assess whether those measurements correlated. In one of the three studies, cortisol and oxytocin remained stable before and after AAI sessions, while HR was lower post-session [36]. In the second, HR, respiratory rate, and cortisol were all higher post-AAI [39]. The third study found higher HR in dogs on AAI days, but no significant cortisol pattern [54]. In contrast, four studies included analyses of both dog behaviors and their cortisol levels, and all found significant correlations between the two [26,42,53,54]. 

Although clear patterns have not yet emerged, three studies listed the dog handlers’ gender identities as a potential confounding variable, with researchers suggesting that male and female handlers could trigger different levels of stress reduction and adaptation in dogs [22,34,54].

## 4. Discussion

### 4.1. Overview of Results

This systematic scoping review analyzed available research in the literature reporting AAIs’ impacts on human and canine stress responses, with a particular focus on molecular biomarkers. We found that AAIs generally reduced human stress levels, as measured by molecular biomarkers, physiological measures, and/or subjective stress responses. However, outcomes frequently varied across measures within a single study.

The evidence in this review also indicates that trained and certified dogs can participate in AAIs without becoming excessively distressed, while the impacts of AAIs on non-certified dogs is unclear. Because few studies reported on dog characteristics, such as breed, sex, size, age, or spay/neuter status, no conclusions could be drawn regarding those traits’ potential impacts on outcomes.

### 4.2. Methodological Issues

The types of AAIs examined in this scoping review varied significantly in length, number, structure, and population, making it difficult to compare outcomes across studies. Further, none of the studies involved broadly represent participant samples across gender, race/ethnicity, and age, thus limiting generalizability.

Most of the included studies only looked at a small number of biomarkers or limited their research to exclusively measuring cortisol, but the studies that did look at multiple measures of stress collectively demonstrated that any single measure provides an incomplete picture of AAIs’ impacts. This is particularly significant when exploring the impacts of AAIs on canine well-being, because so few studies were identified that examined more than one measure of canine stress. Most studies also failed to account for factors known to impact cortisol levels, such as time of day and pre-experimental activities, or to optimize the timing of sample collection based on when cortisol or other stress measures could be expected to peak. This timing varies by the biomarker(s) being studied, but in salivary cortisol—the most commonly studied biomarker in this review—that timing should be 30–45 after the onset of the stressor or intervention [60]. Failure to properly time biomarker data collection could explain the lack of significant results to cortisol responses in several of the reviewed studies and should be avoided in future research.

### 4.3. Limitations

Limitations of this review include restricting screening to the first 100 results in each database, excluding studies that involved children or adults older than 65 years, and only including canine-related AAIs. While the study team’s ability to screen articles in five languages could be considered a strength of the review, there may have been articles published in other languages that were omitted. 

### 4.4. Recommendations for Future Research

This systematic scoping review has provided a comprehensive summary of current research into the effects of AAIs on molecular biomarkers and related measures of stress in dogs and humans. Despite cortisol being the most researched biomarker in studies included in this review, many studies stated that cortisol alone was not a reliable biomarker for measuring stress. A broader array of molecular biomarkers needs to be studied in conjunction with physiological, behavioral, and subjective measures to accurately assess changes in stress and stress responses. In addition, researchers should account for the effects of timing and other confounding variables when undertaking biomarker data collection. 

Generalizability can be improved in future studies by including larger and more diverse participant pools, because stress responses are known to vary according to different factors, including age, sex, gender, and race [6,61,62,63]. In particular, the overrepresentation of English-language articles featuring White women indicates that more research should be conducted by researchers in a variety of contexts. However, the results of several of the included studies indicate that specificity should also be further explored, as preliminary evidence indicates that there may be differences in AAI outcomes across groups. 

Researchers should also continue to explore the impacts of AAIs on canine welfare. While the findings in this review were promising in that most showed a neutral or positive impact of AAIs on canine stress, only nine studies examined this question, most of which used a very limited number of stress-measurement approaches.

## 5. Conclusions

Twenty-seven studies examined the impacts of AAIs on the stress responses of humans and dogs. Overall, the studies indicated that AAIs have positive impacts on human stress responses and do not excessively distress dogs, but specific outcomes varied across and within studies. Because many of the studies had small sample sizes and worked with specific populations, the generalizability of the findings was limited. Future research should use rigorous approaches that include careful timing of sample collection and include multiple approaches to measuring stress-related outcomes.

## Figures and Tables

**Table 1:** Key characteristics of included articles.

Article	Key Demographics	Stressor	Intervention	Stress Measures	Key Outcomes
Barker et al., 2010 [32]	10 healthy adults; 8 female, 2 male; 90% White 6 therapy dogs	Stroop Color Word Test	30-min AAI with own or an unfamiliar (AAA) therapy dog	Biomarkers: Human: sCort, sAA Physiological: Human: BP, HR Subjective: STAI, VAS	sAA, BP, and HR showed little change AAA group showed small increase to sCort with stressor and “negligible” decrease from baseline. Own dog group showed smaller response to stressor and significant decrease post-intervention. Both groups’ subjective stress fell below baseline post-intervention
Barker et al., 2016 [33]	57 adult college students; 44 female, 13 male; 52.6% White 10 therapy dogs	The week before final exams	Control: 15-min attention-control Intervention: 15 min with therapy dog	Biomarkers: Human: sAA, Subjective: PSS, SVAS	No significant pre-post differences to sAA between groups SVAS was lower following intervention: large effect size
Clark et al., 2019 [34]	24 nurses; 23 female, 1 male 4 therapy dogs	AAA stress on dog	AAT visits to outpatient nursing units	Biomarkers: Dogs: sCort	More frequent visits (up to two/week) associated with lower cortisol levels
Clark et al., 2020a [35]	221 adults with fibromyalgia. 93.2% White 19 therapy dogs	Fibromyalgia	Treatment: 20-min AAA with a certified therapy dog and handler Control: 20-min session with handler only	Biomarkers: Humans: sCort, salivary oxytocin Physiological: Humans: Tympanic membrane temperature, HR, HRV Subjective: FIQR, Pain NRS, VAS for “various emotions”	No significant differences between groups in FIQR, NRS VAS, or sCort Treatment group showed significant increase to oxytocin, right tympanic membrane temp, and HRV, and decrease in HR
Clark et al., 2020b [36]	222 adults with fibromyalgia 16 therapy dogs	AAA stress on dog	5 20-min unstructured AAA visits with patients with fibromyalgia	Biomarkers: Dogs: sCort, salivary oxytocin Physiological: Dogs: HR, HRV, tympanic membrane temperature	Dogs showed “neutral to positive response” to AAA sessions. HR and right tympanic membrane temp lower post-session, all other indicators stable
Clark et a., 2020c [26]	9 therapy dog handlers. 8 female, 1 male 9 therapy dogs	Stress on dogs from their first 3 AAT visits to a hospital	1st: walking around hospital 2nd: sitting in a waiting room; people interested in the dog could approach 3rd: 47-min inpatient visit	Biomarkers: Humans: sCort (handlers) Dogs: sCort Subjective: Modified PSS (1–4 scale), Handlers’ rating of dogs’ stress	sCort: Nonsignificant decreases post-visit Handlers’ perceptions of dogs’ stress levels aligned with changes in dogs’ cortisol levels
Coakley et al., 2020 [37]	59 patients; 2 female, 27 male; 93.2% White Therapy dogs	Patients hospitalized in an acute care setting	A 15-min AAT session	Biomarkers: Humans: sCort Physiological: HR, RR Subjective: STAI, Wellbeing VAS, Comfort VAS	Significant improvements in anxiety, comfort and well-being; significant reductions in HR and RR. Nonsignificant changes to cortisol
Cole et al., 2007 [38]	76 adults No details on dogs	Patients with advanced heart failure admitted to a cardiac care or cardiac observation unit of a hospital	Group 1: 12-min hospital visit with a therapy dog Group 2: 12-min visit from a volunteer only Group 3: Usual care	Biomarkers: Humans: Epinephrine and norepinephrine Physiological: Humans: BP; HR; right atrial, pulmonary artery, and capillary wedge pressure; cardiac index; systemic vascular resistance Subjective: STAI	Dog group had lower cardiopulmonary pressures, epinephrine and norepinephrine, and anxiety. Other measures not significantly impacted
De Carvalho et al., 2019 [39]	19 therapy dog handlers, all female 19 therapy dogs	AAA stress on dog	AAI sessions (details varied by team but were typically familiar)	Biomarkers: Dogs: sCort Physiological: Dogs: HR, RR	Dogs had higher HR, RR, and sCort after AAIs than at home, but all HR values were “around the normal range”
Fecteau et al., 2017 [40]	Parents of 114 autistic children Service dogs	Stress related to parenting an autistic child	Service dog or waitlist control	Biomarkers: Humans: sCort Subjective: PSI-SF	Dog group reported reduced parenting stress after 9 months and lower morning cortisol in first 12 weeks
Glenk et al., 2013 [41]	Dog handlers, all female 21 therapy dogs and therapy dogs in training	AAA stress on dog	8 weekly AAIs on-leash or off-leash at three inpatient mental health facilities	Biomarkers: Dogs: sCort	No significant increases in sCort. Off-leash group had lower working cortisol levels than on-leash
Glenk et al., 2014 [42]	Dog handlers 5 therapy dogs	AAA stress on dog	5 weekly AAAs at an inpatient substance abuse treatment facility	Biomarkers: Dogs: sCort	sCort decreased post-session, with significant decreases in last 2 sessions. No significant difference in sCort between working and nonworking days
Haubenhofer and Kirchengast, 2007 [22]	13 dog handlers; 12 female, 1 male 18 therapy dogs	AAA stress on dogs and handlers	AAT sessions over 3 months (details varied by handler-dog team)	Biomarkers: Humans; sCort (handlers) Dogs: sCort Subjective: Emotion questionnaire	Handlers and dogs had higher sCort on AAT days compared to control days In handlers, sCort increased steadily with session duration; in dogs, with number of sessions/week
Kline et al., 2020 [43]	122 emergency medicine providers; 86.8% White	Occupational stress of emergency medicine providers	Group 1: no intervention Group 2: 5 min coloring Group 3: 5-min AAI	Biomarkers: Humans: sCort Subjective: SVAS, PSS-10, FACES stress scale	SVAS showed reduction in stress in dog group, but PSS-10 did not. sCort decreased significantly in both coloring and dog groups compared to control.
Koda et al., 2016 [44]	78 inmates in a Japanese men’s prison 48 therapy dogs	Stress related to imprisonment. Many also had psychiatric and/or developmental disorders	12 weekly, 70-min group AAT session	Biomarkers: Humans: sCort Subjective: Mood questionnaire	35% reported mood improvements after AAT; 6% mood reductions Inmates with psychiatric but not developmental disorders showed decreased sCort post-AAT; inmates with both types of disorders or developmental disorders only did not show significant changes
Krause-Parello et al., 2018 [45]	25 military veterans; 21 male, 4 female; 68% White 1 facility dog	Hospitalized veterans being seen by a palliative care psychologist	Group 1: 20-min AAT visit with a psychologist Group 2: 20-min psychologist visit only	Biomarkers: Humans: sCort, sAA, IgA Physiological: Humans: BP, HR Subjective: Coping Strategy Indicator, Seeking Support subscale; CDC Health-Related Quality of Life; UCLA Loneliness Scale; PSS	Significant decreases in sCort and HR in both groups, dog group showed lower HR than psychologist-only group sAA and IgA not significantly different between conditions
Krause-Parello et al., 2019 [46]	120 patients; 95 male, 25 female; 59.1% White Therapy dogs	Military personnel who had recently been aeromedically evacuated	Group 1: 20-min AAI Group 2: 20-min info session about assistance dogs	Biomarkers: Humans: sCort, sAA, IgA Subjective: PTSSS, PCL-M	sCort decreased significantly in the AAI group compared to control group Patients in experimental condition with higher PTSSS had greater reduction in stress as assessed by IgA No significant difference in sAA between groups
Krause-Parello et al., 2020 [47]	33 military veterans; 26 male, 7 female; 75.8% White Shelter dogs	Military veterans	Group 1: 4 30-min weekly dog walks Group 2: 4 30-min weekly walks with another human	Biomarkers: Humans: sCort, sAA Physiological: Humans: HRV Subjective: PCL-M, PSS	Walking with a dog or another person led to decreases in sCort among those with low PTSD symptom severity, but sAA did not change significantly Individuals with high PTSD symptoms did not show significant change to sAA in dog walk group, but did in human walk group. In this group, average HRV increased in dog walk group but decreased in human walk group
Lass-Hennemann et al., 2014 [48]	80 healthy female university students Therapy dogs	11 min “trauma film” with fictional scenes of physical and sexual violence	Watched film with: Group 1: therapy dog Group 2: toy dog Group 3: friendly person Group 4: alone	Biomarkers: Humans: sCort Physiological: Humans: BP, HR Subjective: STAI, PANAS	Dog group showed lower STAI and PANAS scores than toy dog or alone groups, and similar to friendly human group No significant differences in physiological or sCort stress between groups
Lass-Hennemann et al., 2018 [49]	60 healthy female university students Therapy dogs	11 min “trauma film” with fictional scenes of physical and sexual violence	After film: Group 1: Interacted with a friendly dog for 15 min Group 2: Watched a film clip showing a person interacting with a friendly dog Group 3: Told to relax	Biomarkers: Humans: sCort Physiological: Humans: BP, HR Subjective: STAI, PANAS, BDI-II, record of intrusive thoughts and distress	Dog group reported less anxiety, and more positive and less negative affect, but had smaller decrease in physiological arousal after film, compared to other groups. No differences in intrusive thoughts between the groups
Machová et al., 2019 [50]	22 female nurses; 13 worked in rehabilitation and physical medicine (PRM), 9 worked in internal medicine and long-term care 1 therapy dog	Occupational stress of nurses	Condition 1: normal work, no break Condition 2: normal work, break of choice Condition 3: normal work, AAT break	Biomarkers: Humans: sCort	sCort levels of PRM nurses did not decrease after AAT, but did in those working in internal medicine; likely due to low initial cortisol levels from PRM nurses “Break of choice” groups did not show decrease in sCort
Menna et al., 2019 [51]	10 dialysis patients; 7 male, 3 female, with comparable stage of renal damage and “relational difficulties” 1 therapy dog	Dialysis patients affected by end-stage renal disease	11 weekly hour-long AAA sessions	Biomarkers: Humans: serotonin, oxytocin	No significant changes to serotonin before and after session, but serotonin and oxytocin increased from one session to the next
Nepps et al., 2014 [52]	218 patients, relatively balanced between men and women (exact details not shared to protect privacy) 80% of sessions occurred with the same female border collie; other details not provided	Patients hospitalized in a mental health unit	Group 1: 1-h AAA session Group 2: 1-h stress management program	Biomarkers: Humans: sCort Physiological: Humans: BP, pulse Subjective: Burns Depression Checklist, Burns Anxiety Inventory, 0–10 pain scale	Significant decreases in depression, anxiety, pain, and pulse after AAA, comparable to those in the traditional stress management group. No changes in BP and sCort
Ng et al., 2014 [53]	16 therapy dog handlers; 2 male, 14 female 15 therapy dogs	AAA stress on dog	Setting 1: 60-min AAA with college students Setting 2: 60 min in novel room near a stranger Setting 3: 60 min of normal activity at home	Biomarkers: Dogs: sCort	sCort levels significantly higher in novel setting compared to AAA or home settings. sCort not statistically different between AAA and home settings
Pirrone et al., 2017 [54]	4 female therapy dog handlers 4 therapy dogs	Familiar AAA stress on dogs and handlers	5 weekly, 55-min AAAs with 2–5 adults Control: HR and saliva collected at similar times of day from home	Biomarkers: Humans: sCort Dogs: sCort Physiological: Humans; HR Dogs: HR	Handlers’ sCort levels decreased over time during both activity and control days. Dogs showed similar pattern, but it was not statically significant. No difference in handlers’ sCort levels on AAA compared to control days. Dogs’ HR was higher during AAA days than in control days
Polheber and Matchock, 2014 [12]	48 university students; 26 males; 64% White 1 therapy dog, female Golden Retriever	TSST	TSST alone, with a human friend, or with a novel dog	Biomarkers: Humans: sCort Physiological: Humans: HR Subjective: STAI	Participants’ sCort levels were lower with dogs’, as compared to with a friend or alone. STAI responses not associated with sCort, but HR was
Rodriguez et al., 2018 [55]	73 post-9/11 military veterans with PTSD; 59 male 45 service dogs	Veterans with PTSD	Service dog. Both groups continued to receive usual care.	Biomarkers: Humans: sCort Subjective: PCL, PROMIS, PSQI	Participants with a service dog showed higher cortisol awakening response and reported lower anxiety, anger, and sleep disturbance, and less alcohol abuse, compared to waitlist controls

Key: BDI = Beck Depression Inventory, BP = blood pressure, FIQR = Fibromyalgia Impact Questionnaire—Revised, HR = heart rate, HRV = heart-rate variability, IgA = Immunoglobulin A, NRS = numeric rating scale, PANAS = Positive and Negative Affect Schedule, PCL-M = PTSD Checklist—Military Version, PSQI = Pittsburgh Sleep Quality Index, PSI-SF = Parenting Stress Index—Short Form, PSS = Perceived Stress Scale, PTSSS = post-traumatic stress symptom severity, PROMIS = Patient-Reported outcome measurement information system, RCT = Randomized control trial, RR = respiratory rate, sAA = salivary alpha-amylase, sCort = salivary cortisol, sNGF = salivary nerve growth factor, STAI = State–Trait Anxiety Inventory, SVAS = Stress Visual Analog Scale, TSST = Trier Social Stress Test, VAS = Visual Analog Scale.

## Data Availability

Not applicable.
